# The Vasoactive Mas Receptor in Essential Hypertension

**DOI:** 10.3390/jcm9010267

**Published:** 2020-01-18

**Authors:** Amalie L. Povlsen, Daniela Grimm, Markus Wehland, Manfred Infanger, Marcus Krüger

**Affiliations:** 1 Department of Biomedicine, Aarhus University, Høegh-Guldbergsgade 10, 8000 Aarhus C, Denmark; amaliepovlsen@hotmail.com; 2 Clinic for Plastic, Aesthetic and Hand Surgery, Otto von Guericke University, Leipziger Str. 44, 39120 Magdeburg, Germany; markus.wehland@med.ovgu.de (M.W.); manfred.infanger@med.ovgu.de (M.I.); marcus.krueger@med.ovgu.de (M.K.)

**Keywords:** hypertension, renin-angiotensin-aldosterone system, MasR, angiotensin-(1-7)

## Abstract

The renin–angiotensin–aldosterone system (RAAS) has been studied extensively, and with the inclusion of novel components, it has become evident that the system is much more complex than originally anticipated. According to current knowledge, there are two main axes of the RAAS, which counteract each other in terms of vascular control: The classical vasoconstrictive axis, renin/angiotensin-converting enzyme/angiotensin II/angiotensin II receptor type 1 (AT_1_R), and the opposing vasorelaxant axis, angiotensin-converting enzyme 2/angiotensin-(1-7)/Mas receptor (MasR). An abnormal activity within the system constitutes a hallmark in hypertension, which is a global health problem that predisposes cardiovascular and renal morbidities. In particular, essential hypertension predominates in the hypertensive population of more than 1.3 billion humans worldwide, and yet, the pathophysiology behind this multifactorial condition needs clarification. While commonly applied pharmacological strategies target the classical axis of the RAAS, discovery of the vasoprotective effects of the opposing, vasorelaxant axis has presented encouraging experimental evidence for a new potential direction in RAAS-targeted therapy based on the G protein-coupled MasR. In addition, the endogenous MasR agonist angiotensin-(1-7), peptide analogues, and related molecules have become the subject of recent studies within this field. Nevertheless, the clinical potential of MasR remains unclear due to indications of physiological-biased activities of the RAAS and interacting signaling pathways.

## 1. Introduction

Hypertension (HT) affects more than 1.3 billion individuals worldwide and is regarded a major risk factor for mortality and morbidity [1,2]. Extensive research confirms that HT is an independent risk factor for severe cardiovascular (CV) and renal events including myocardial infarction, heart failure, ischemic stroke, peripheral artery disease, and end-stage renal disease [3,4], and represents a major public health problem.

According to the guidelines of the European Society of Cardiology (ESC) and the European Society of Hypertension (ESH), HT is defined as an office systolic blood pressure (BP) ≥ 140 mmHg and/or diastolic BP of ≥ 90 mmHg [3]. This definition was generally accepted until 2017, where the guidelines of the American College of Cardiology (ACC) and the American Heart Association (AHA) lowered the threshold for diagnosis of HT to a systolic BP of ≥ 130 mmHg and/or a diastolic BP of ≥ 80 mmHg [4].

HT is considered a multifactorial disease that involves environmental and genetic factors, as well as lifestyle and risk-conferring behaviors [5,6]. It can be categorized as essential or secondary HT, of which essential HT accounts for about 95% of the hypertensive population, and refers to HT with no identifiable cause [3,7], while secondary HT accounts for 5–15% [3] and represents a complication to an identified cause, e.g., renovascular disease, endocrine disorders or drug association [3]. In comparison, proper and early intervention in secondary HT may help control BP in addition to treating the underlying cause [3], while the polygenic character of essential HT complicates treatment [8]. In addition, experimental research has suggested a link between HT and structural alterations in genes encoding separate components of the renin-angiotensin-aldosterone system (RAAS) [9,10], indicating that changes in the activity of RAAS-regulated genes may increase the risk of developing HT [8]. These findings suggest that HT is associated with a dysfunctional RAAS.

The RAAS has been studied extensively, and is known to play a pivotal role in BP control [11]. Among current treatment strategies, RAAS targeting is common and includes renin inhibitors, angiotensin-converting enzyme (ACE) inhibitors, angiotensin II type 1 receptor (AT_1_R) antagonists (also known as angiotensin II receptor blockers, ARBs), mineralocorticoid receptor-antagonists and aldosterone synthase inhibitors, which all serve to block the RAAS at different loci [12,13]. In addition, diuretics, β-adrenoceptor antagonists and calcium channel blockers are drug classes that aid in lowering both BP and risk of CV events [3]. According to available data, the activity of the RAAS depends on a balance between the classical vasoconstrictive axis and the opposing vasorelaxant axis [14]. The latter is said to counteract the adverse effects mediated by AT_1_R, which is linked to the pathophysiological actions of angiotensin II (Ang II) and to cardiovascular disease (CVD) [14]. The beneficial effects of the G-protein coupled Mas receptor (MasR) was originally associated with binding of endogenous Ang-(1-7). Yet, the discovery of novel agonists, e.g., CGEN-856S and AVE 0991, has extended the therapeutic potential of the RAAS, and furthermore, encouraging research data has led to the investigation of the potential of MasR-based drugs [15].

By illuminating various actions and interactions of MasR, related to vasculature and BP regulation, this review aims to examine the role of MasR in HT and to evaluate its potential as a target in antihypertensive drugs.

## 2. The Renin-Angiotensin-Aldosterone System (RAAS)

### 2.1. The Classical RAAS

Renin is considered the rate-limiting component of the RAAS, in which it is released in response to stimuli related to lowered BP, e.g., a decreased renal perfusion pressure, an increased sympathetic tone or a decreased delivery of sodium chloride to the macula densa [9]. Renin cleaves hepatic angiotensinogen into angiotensin I (Ang I), which is then converted into the main effector molecule, Ang II, by the angiotensin-converting enzyme (ACE) [13]. Ang II acts through G-protein coupled AT_1_R to promote water and sodium retention, vasoconstriction, pro-inflammation, and adrenal secretion of aldosterone [13], which further synergistically accelerates renal sodium and water retention by stimulating the mineralocorticoid receptors [11]. In addition, the activation of mineralocorticoid receptors in extra-renal tissues, e.g., the heart and vessels, has been reported to promote endothelial dysfunction and tissue remodeling due to formation of reactive oxygen species (ROS) [12,13].

Ang II also binds to angiotensin II type 2 receptor (AT_2_R) of the proximal tubule, cortical collecting ducts and resistance arteries, which promotes hypotensive and natriuretic effects, thus opposing the actions of AT_1_R [15,16]. However, data suggest that AT_1_R predominates in a physiological setting, due to the fact that experimental activation of AT_2_R often requires pretreatment with ARBs, to prevent Ang II from binding AT_1_R instead [16]. Local actions of Ang II thus depend on the combined net effect of AT_1_R and AT_2_R [17], and it seems likely that an abnormally high activity in the AT_1_R predisposes to HT.

### 2.2. Extension of the RAAS

**Figure 1:**
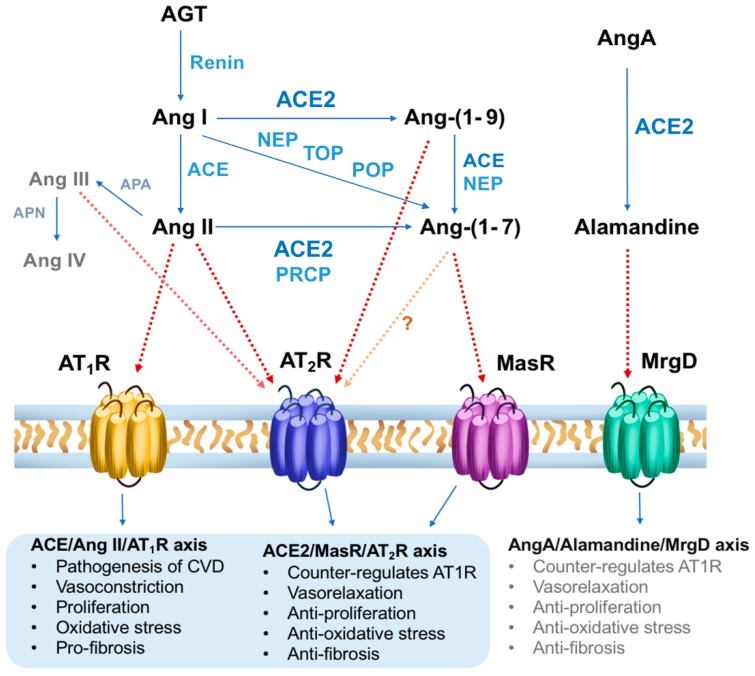
Schematic overview of the renin/ACE/Ang-II/AT_1_R axis, ACE2/MasR/AT_2_R axis and AngA/Alamandine-MrgD axis, modified from [22]. ACE, Angiotensin converting enzyme; AGT, angiotensinogen; Ang, angiotensin; APA, aminopeptidase A; APN, aminopeptidase N; AT_1_R, angiotensin II type 1 receptor; AT_2_R, angiotensin II type 2 receptor; MasR, Mas receptor; MrgD, Mas-related G-protein coupled receptor type D; NEP, neutral endopeptidase, POP, prolyloligopeptidase; PRCP, prolylcarboxypeptidase; TOP, thimet oligopeptidase.

In the past few decades, extensive research on the RAAS has expanded our general understanding of the system, making it clear that it is much more complex than originally anticipated [15] and comprises various biologically active metabolites (Figure 1).

An alternate substrate for Ang II generation is Ang-(1-12), which serves as an upstream precursor for Ang I and Ang II [18]. Cleavage of Ang II by aminopeptidase A (APA) generates Ang-(2-8) (Ang III), which binds AT_1_R to promote a pressor response similar to that of Ang II, but with a prominent role in the brain [19]. Specific APA inhibitors such as RB150 have thus been developed and are still being tested in animals [20]. Further cleavage of Ang III produces Ang-(3-8) (Ang IV), which binds AT_4_R to promote vasodilation in cerebral and renal vascular beds, facilitating renal blood flow and sodium excretion [21].

Ang-(1-9) is another interesting metabolite due to its anti-hypertensive actions. It is generated from Ang I and induces AT_2_R-mediated vasodilation, natriuresis, decreased CV remodeling and anti-proliferation [23]. In hypertensive models, Ang-(1-9) has been shown to ameliorate CV injury, while in clinical studies, it opposed CV remodeling in patients with HT and/or heart failure [24]. Evidence thus suggest a therapeutic potential of Ang-(1-9), but an in-depth evaluation is considered beyond the scope of this review.

Heptapeptide Ang-(1-7), which is a specific MasR agonist, plays a central role in the counterregulatory arm of the RAAS, ACE2/Ang-(1-7)/MasR. It can be generated by different enzymes, but with the most potent being ACE2 [25], which can produce Ang-(1-7) through hydrolysis of Ang II, or from Ang I with Ang-(1-9) as an intermediate step [15]. The former pathway is more favorable, due to ACE2′s affinity to Ang II [17]. Ang-(1-7) only differs from Ang II in its lack of the C-terminal phenylalanine residue, and it was considered as a MasR agonist upon the discovery of a selective Ang-(1-7)-antagonist [26,27]. Ang-(1-7) exerts nitric oxide (NO)-dependent vasorelaxation via the MasR, which opposes the effects of AT_1_R [26]. ACE2 and MasR are expressed in the same tissues, suggesting that Ang-(1-7) provides specific protection in these organs [25]. Yet, the relative role of different enzymes in the synthesis of Ang-(1-7) vary considerably, depending on cell type and tissue [28].

Furthermore, recent studies revealed interactions of Ang-(1-7) with other RAAS receptors, including AT_1_R and AT_2_R, suggesting that the actions of Ang-(1-7) rely on an interplay with the classical RAAS, and additionally depend on local expression levels of MasR, Ang-(1-7), Ang II, AT_1_R and AT_2_R [29].

Lastly, it should be mentioned that ACE2 converts angiotensin A into vasoactive alamandine, which is related to Ang-(1-7) and binds a Mas-related G-protein coupled type D receptor (MrgD) [30]. This constitutes an AngA/alamandine-MrgD pathway with similar properties to the ACE2/Ang-(1-7)/MasR axis [20]. The comparative difference is investigated in preclinical studies [31].

### 2.3. Blood Pressure Regulation by the RAAS

BP is a measure to describe the intravascular pressure generated by circulating blood, and it can be calculated from cardiac output (CO) and total peripheral resistance (TPR), in accordance with the law of Ohm [32]. Both CO and TPR, are affected by various mechanisms, including the RAAS, which is a vital regulator of homeostasis [25] and in particular of fluid volume, BP, electrolyte balance, and neuroendocrine functions [9].

According to the classical paradigm [15], sodium reabsorption is mediated by Ang II acting on renal sodium-hydrogen exchanger 3, while aldosterone acts on sodium-chloride cotransporter, ENaC, and renal outer medullary potassium channel [15]. Salt and water retention is triggered by hypovolemia, while potassium excretion is additionally stimulated by hyperkalemia [15]. As a consequence, BP is increased due to an expansion in the extracellular fluid volume. The RAAS-mediated pressor effect is further accelerated by an increase in sympathetic activity and a secretion of aldosterone and vasopressin [5,17]. With these mechanisms in consideration, the risk of developing essential HT is increased with a hyperactive RAAS. This theory is being supported by the successful treatment with RAAS blockers [9].

### 2.4. The Two Arms of the RAAS

The classical pathways leading to generation of angiotensin peptides have been described extensively, but the general understanding was challenged by the first description of Ang-(1-7) as an endogenous inhibitor of the RAAS [33]. This has led to a growing interest in characterizing counterregulatory components of the RAAS, while searching for novel targets in CVD therapy [12]. It is now believed that the RAAS consists of two distinct, opposing arms: ACE/Ang II/AT_1_R and ACE2/Ang-(1-7)/MasR.

Overstimulation of the vasoconstrictive axis is associated with CVD, including HT and myocardial infarction with possible progression to heart failure [34]. The pathophysiology is associated with excessive vasoconstriction, fibrosis, endothelial dysfunction, tissue remodeling [34], and in particular with ROS formation by NADPH oxidase [35]. In addition, research has indicated that the detrimental effects are partially associated with a suppression of the counterregulatory arm of the RAAS, resulting in increased levels of Ang II and reduced levels of Ang-(1-7) [36]. Taken together, blocking the classical RAAS has served an effective the strategy in pharmacotherapy for decades.

On the other hand, the alternative vasorelaxant axis constitutes an intrinsically protective mechanism, associated with beneficial outcomes in CVD [25]. The actions of Ang-(1-7) were originally thought to be mediated exclusively through MasR. However, it has been demonstrated that Ang-(1-7) also binds to AT_2_R and AT_1_R, when present in higher levels [37,38]. In fact, it has been proposed that the AT_2_R is responsible for the vasodepressor effects of Ang-(1-7) [38], while other studies suggest that Ang-(1-7) inhibits AT_1_R in a non-competitive manner [31].

MasR is a biologically active peptide encoded by the *MAS1* gene, which was identified as a proto-oncogene, based on its ability to induce tumorigenicity in murine cells [26]. MasR is predominantly expressed in the brain and the testes, while moderate levels are found in the heart, kidney and vessels [39]. It has a similar structure to other G-protein coupled receptors (GPCRs) [40]. Human endothelium express MasR, through which Ang-(1-7) alters local redox balance and promotes vasodilation, oxidative stress reduction and antifibrosis [39]. Based on the observation that it triggered the release of vasopressin in a similar manner to Ang II, Ang-(1-7) was originally thought to be a selective MasR agonist [5,33]. The endothelial synthesis of Ang-(1-7) was first described by Santos et al. [41].

MasR was shown to constitutively couple to G_q_-proteins and to promote ischemia-reperfusion in rats stimulated with synthetic peptide ligands [42]. Controversially, Ang-(1-7) does not induce G_q_-related alternations, but rather acts through a non-G-protein mechanism to promote release of arachidonic acid, bradykinin and prostaglandins, while additionally activating endothelial nitric oxide synthase (eNOS) [42]. This was observed in spontaneously hypertensive rats (SHRs), in which the protective axis was blocked with an Ang-(1-7) antagonist, A-779, repealing the effects of Ang-(1-7) [35,43]. In a similar manner, this was observed in mice with ablation of the *MAS1* gene [39]. Yet, a study on human aortic endothelial cells suggested that Ang-(1-7) attenuates the classical RAAS through a mitogen-activated protein kinase cascade [35], while another proposed mechanism was that Ang-(1-7) inhibits Ang II-induced c-Src phosphorylation, which increases NO bioavailability and attenuates ROS formation [35]. Moreover, the ACE2/Ang-(1-7)/MasR axis could be a promising therapeutic option for diabetic patients since the activation of this axis through the use of cyclic Ang-(1-7) offered renoprotection in mice with type 2 diabetic nephropathy [44].

Taken together, these data indicate that distinct CV actions of Ang-(1-7) are mediated through MasR [15], but that this involves an interplay with other pathways of the RAAS. Furthermore, the effects of Ang-(1-7) seem to be affected by factors such as the presence of additional receptors and angiotensin peptides, local expression levels, and the general state of the tissue [17].

## 3. Novel MasR Agonists

In recent decades, the protective arm of the RAAS has been considered a promising approach in treatment of CVD, and different strategies are under investigation.

Based on the knowledge of ACE2, which is crucial in maintaining the balance between the opposing RAAS axes, a novel therapeutic strategy aims to increase endogenous levels of Ang-(1-7) by using ACE2 activators [45]. Alternatively, injection of endogenous alamandine has been shown to reduce BP and decrease post-ischemic reperfusion injury in SHRs [30], which is likely because of the morphological similarity between alamandine and Ang-(1-7). However, alamandine does not bind to MasR but to the MrgD receptor with similar properties [31]. Furthermore, alamandine has been compared with synthetic AVE 0991, which was the first orally active MasR agonist, in which organ protection was demonstrated as a dose-dependent vasorelaxation in aortic rings of SHRs [43]. This effect was absent in MasR deficient mice [46], and Faria-Silva et al. [47] further demonstrated how Ang-(1-7) and AVE 0991 both potentiate vasodilation in Wistar rats. The effects of AVE 0991 are multiple and quantitatively comparable to those of Ang-(1-7) [25]. In addition, blockage of AVE 0991 with Ang-(1-7) antagonists, A-779 and d-Pro^7^-Ang-(1-7), suggest that at least some of its actions are mediated through MasR [48]. So far, AVE 0991 has not yet been studied in humans [20].

Another strategy is the generation of metabolically stable Ang-(1-7) analogues [49,50] by encapsulating them with hydroxypropyl-β-cyclodextrin which protects the peptides from digestive enzymes, thus producing a feasible formulation for oral administration of Ang-(1-7) [50]. Another example is the peptidase-resistant thioether-bridged Ang-(1-7), which was shown to exert vasorelaxation in aortic rings of rats in addition to improving cardiac remodeling and endothelial function following myocardial infarction [51].

Advances in biotechnology have further enabled the discovery of novel agonists, such as CGEN-856 and CGEN-857 [28]. These were reported to display specificity for MasR, without binding neither to AT_1_R nor to AT_2_R. Vasoprotective effects of CGEN-856S were demonstrated in animals and included a dose-dependent vasodilation of precontracted aortic rings (maximal value 39.99 ± 5.03%) and vasorelaxation observed with infusion in SHRs [28].

Taken together, publications on novel MasR agonists report various beneficial properties of the receptor, while application of MasR-blockers, which abolish the actions of Ang-(1-7) and related peptides, additionally supports the theory that MasR improves endothelial function. Furthermore, genetic modification of animal models, e.g., Mas-deficient mice, emerged as a valuable tool in assessing actions and interactions of MasR, yielding an increasing body of evidence that reinforces the clinical potential of MasR in the management of HT.

## 4. Mas Receptor in the Management of Hypertension

Within the past decade, there seems to be an ongoing shift in the strategy of interfering with the activity of the RAAS [52]. Rather than blocking the classical pathway, as extensively done with the established drugs, an increasing body of evidence on the beneficial effects of MasR has encouraged researchers to exploit the potential of the ACE2/Ang-(1-7)/MasR axis in CV therapy [25,52]. This can be done using various Ang-(1-7) analogues and MasR agonists, as described (Table 1).

Notably, data on the competitive antagonism between Ang-(1-7) and Ang II for AT_1_R has resulted in contrasting results and binding controversies, suggesting the possibility of pluridimensional receptor actions, which needs further clarifications. In order to identify the relevance of an AT_1_R/Ang-(1-7) axis Galandrin et al. [29] studied the interactions of Ang-(1-7) and AT_1_R by comparing Ang-(1-7)-mediated effects in phenylephrine-precontracted aortas in wild-type mice with those in AT_1_R knockout mice. They found that Ang-(1-7) attenuated the contraction in wild type, but not in knockout or in the presence of AT_1_R antagonist candesartan. The results demonstrated the possibility that Ang-(1-7) shows biased agonism for AT_1_R, or even antagonism, independent of MasR. In a similar experimental setting with an Ang II-mediated aortic precontraction, Ang-(1-7) conversely potentiated the contraction through MasR and thus promoted the development of HT [29].

Taken together, these studies demonstrate biased actions of Ang-(1-7) and suggest that the effects mediated by the protective arm of the RAAS may depend on local interactions with the classical system [53]. Evidence of interactions between MasR and additional receptors, including bradykinin and endothelin receptors, further complicate the understanding of GPCR Mas [54,55], stressing the need for future studies to clarify how the relationship between MasR and Ang-(1-7) depends on e.g., cell type and local expression of other GPCRs. This could be crucial for developing future drugs targeting the MasR.

**Table 1:** Emerging potential drugs interacting with MasR.

Drug	Mechanism of Action	Effects on Blood Pressure	Status
AVE 0991	AT_2_R/MasR agonist. Orally active nonpeptide drug.	AVE 0991 binding to bovine aortic endothelial cell membranes [43]. ACh-induced vasorelaxation in rats [47]. Potentiation of bradykinin through a Mas-mediated mechanism [48].	Preclinical studies
CGEN-856S CGEN-857	MasR agonist. Peptide drug.	Vasorelaxation in murine aortic rings [28]. Dose-dependent decrease in mean arterial pressure (MAP) in SHRs [28].	Preclinical studies
HPβCD- Ang-(1-7)	Stable Ang-(1-7) analogue. Hydroxypropyl-β-cyclodextrin protects Ang-(1-7) from digestive tract enzymes.	Chronic oral administration lowers BP in rats following ischemia-reperfusion injury [56].	Preclinical studies
Cyclic Ang-(1-7)	Peptidase resistant Ang-(1-7) analogue.	Improves endothelial function post-MI in male Sprague Dawley rats [51]. cAng-(1-7) improved peripheral endothelium-dependent vasodilation, as measured in isolated aortic rings [51].	Preclinical studies
RB150/QGC001	A central acting prodrug of the selective APA inhibitor, EC33. Orally available compound with the ability to cross the blood brain barrier.	Dose-dependent and long-lasting reduction in BP in rats, possibly following a specific blockage the of brain renin–angiotensin–aldosterone system [57].	Preclinical and phase II studies
Alamandine	Vasoactive peptide derivative of AngA with selective agonism on MrgD.	Central and peripheral BP reduction [20]. Diminishes reperfusion injury after ischemia [30].	Preclinical studies

## 5. Discussion

HT is a major risk factor for CV morbidity and mortality, and extensive research has been conducted to elucidate the pathophysiology of essential HT, which yet remains unclear [1]. Nevertheless, current data emphasizes that the NO-pathway plays a crucial role and that there is a distinct link between HT and a dysfunctional RAAS [9,10].

Within the past years, research in the field of antihypertensive therapy includes alternative RAAS pathways, novel mineralocorticoid receptor-antagonists, renin inhibitors and aldosterone synthesis inhibitors, peripheral noradrenergic inhibitors and gastrointestinal sodium modulators. This review particularly examined the clinical potential of MasR, which ameliorates CV injury by increasing levels of NO while reducing levels of ROS [35].

As demonstrated in animal-based studies, knowledge about the protective arm of the RAAS may pave the way for a new direction in treatment of HT, focusing on the endogenous protective mechanisms of the RAAS, rather than on the blockage of the classical system [52]. The potential of a new direction is reinforced by the positive CV effects of MasR agonists, such as AVE 0991, CGEN-856, or chemically modified Ang-(1-7). In preclinical studies it was shown that the nonpeptide compound AVE 0991 efficiently mimics the effects of Ang-(1-7) on the endothelium. The oral drug acts the stimulation of a specific, endothelial Ang-(1-7)-sensitive binding site causing kinin-mediated activation of endothelial NO synthase in bovine aortic endothelial cells [43]. Short-term Ang-(1-7) infusions significantly elevated the hypotensive effect of an intra-arterial ACh administration in normotensive rats [47]. A similar effect was seen with its analogue AVE 0991 [47]. The knowledge that Ang-(1-7) and its analogues improve endothelial function in vivo. opens new possibilities for the future treatment of CVD such as hypertension, and heart failure [47]. In addition, a synergistic effect of AVE 0991 and bradykinin on NO release was measured in normotensive rats. These findings show that AVE 0991 potentiates bradykinin through an Ang-converting enzyme–independent, NO-dependent receptor Mas-mediated mechanism. This effect may contribute to the improvement of endothelial function by AVE 0991 in vivo. [48].

Moreover, CGEN-856 and CGEN-857 revealed a high specificity for the Mas receptor, eliciting calcium influx in Chinese hamster ovary cells overexpressing Mas [28]. These peptides activate G protein–coupled receptors. CGEN-856S induced a NO- and Mas-dependent vasorelaxation in isolated aortic rings and decreased MAP in spontaneous hypertensive rats (SHRs) [28]. CGEN-856S is a novel Mas agonist with a therapeutic value, because it induces vasorelaxant, antihypertensive, and cardioprotective effects in SHRs [28]. There are similarities and differences among CGEN-856S, Ang-(1-7), and AVE 0991. CGEN-856S is apparently more stable than Ang-(1-7) and has no ACE-inhibitory activity. Due to its hydrophobicity, AVE 0991 is able to cross the blood-brain barrier and also shows central actions [28]. Some central effects of Ang-(1-7) are opposite to those evoked in the periphery. Therefore, it is important to study the possibility that some peripheral effects of AVE 0991 could be masked by its central actions. On the other hand, Ang-(1-7) is endogenous and has demonstrated well-characterized Mas-dependent actions [28].

Moreover, the inclusion of Ang-(1-7) into the oligosaccharide hydroxypropyl β-cyclodextrin (HPβCD) provided a stable oral analogue [56]. Long-term treatment with HPβCD/Ang-(1-7) in rats could attenuate the pathological remodeling process post-myocardial infarction (MI). This data indicated that Ang-(1-7) is acting beneficial in CVD [56].

In addition, a further compound is the stabilized, thioether-bridged analogue of Ang-(1-7) called cyclic Ang-(1-7), which was tested in a rat model of MI [51]. cAng-(1-7) lowered left ventricular end-diastolic pressure and improved endothelial function in rats. A recent paper showed that adding cAng-(1-7) to ACE-inhibitor therapy is beneficial to diabetic patients not completely responding to ACE-inhibitor therapy [44].

Another aspect to discuss is that a hyperactive brain renin angiotensin system (RAS) is involved in the development and maintenance of HT in several hypertensive animal models. In the murine brain, APA is involved in the conversion of Ang II to Ang III, an effector peptide of the brain RAS and responsible for vasopressin release [57]. RB150 is a prodrug of the specific and selective APA inhibitor EC33. Intravenously administrated RB150 is crossing the blood-brain barrier, inhibits brain APA, and blocks central Ang III formation in rats [57]. These findings demonstrate an important role of brain APA as a candidate target for the treatment of HT. The authors suggest that RB150, a potent systemically active APA inhibitor, could be the prototype of a new class of antihypertensive agents for the treatment of certain forms of HT [57]. Targeting Ang III by inhibiting brain APA is now considered a novel treatment strategy in HT [58]. After crossing the blood-brain barrier RB150 generates two active molecules of EC33 that block brain APA activity. RB150 reduced blood pressure through inhibition of vasopressin release, which elevated diuresis. This is followed by a reduction in extracellular volume, a decrease in sympathetic tone, leading to a reduction of vascular resistances, and the improvement of the baroreflex function [58].

Today, RB150 received the new name firibastat by the World Health Organization. Phase Ia/Ib clinical trials demonstrated that firibastat is clinically and biologically well tolerated in healthy volunteers [58]. The clinical efficacy of firibastat in hypertensive patients was investigated in two phase II studies. Firibastat could represent the first drug of a novel class of antihypertensive drugs targeting the brain RAS [58].

New therapeutic approaches to prevent the activation of the brain neuromodulatory pathway may lead to improve heart failure (HF) are under investigation. The phase II study ‘QUantum Genomics Incremental Dosing in Heart Failure - QUID-HF’ (QUID-HF; ClinicalTrials.gov Identifier: NCT02780180) investigates safety and efficacy of the drug QGC001 (APA inhibitor and prodrug of EC33) vs. placebo in heart failure patients. Current phase III studies on HT with this drug are in the planning phase. Quantum Genomics announces positive FDA feedback on phase III program design of firibastat (QGC001) in resistant arterial hypertension.

Furthermore, ACE2 also converts Angiotensin A (AngA), a product of Ang II, into the peptide Alamandine, which interacts with the Mas-related G protein-coupled receptor D (MrgD) [20]. Alamandine decreased like AVE 0991 ventricular hypertrophy, reduced blood pressure and decreased reperfusion injury after ischemia in animal models [30,59,60,61].

The interactions of alamandine and Ang-(1-7) with the MrgD receptor demonstrate a potential role of this pathway in cardiovascular pathophysiology and diseases. This novel molecular pathway may play a key role in peripheral and central BP regulation and the process of cardiovascular remodelling.

Taken together, studies on Ang-(1-7) suggest the possibility of pluridimensional receptor actions, which depend on an interplay with additional pathways of the RAAS [53]. ACE2 and Ang-(1-7) counteract the adverse effects of RAS components (Figure 1) and have demonstrated protective effects on the vascular and cardiovascular system. Randomized clinical trials should be performed to study the effects of therapies targeting Ang-(1-7) and the Mas receptor. In addition, available data support the hypothesis that MasR acts to maintain endothelial function, as seen in MAS deficient mice or with application of MasR blockers [29].

## 6. Conclusions

It has become evident that the RAAS is more complex than originally anticipated and that an abnormally high activity within the system, for instance due to an imbalance between the counterregulatory axes, may contribute to the development of HT. In several preclinical studies, it has been demonstrated that amplification of the protective arm opposes the deleterious CV effects of the classical RAAS, by decreasing local tissue formation of ROS while stimulating the release of powerful locally acting vasodilators: NO, bradykinin and prostaglandins. Moreover, the pathophysiological consequences of the RAAS seem to depend on the balance obtained within the system, varying with changes in tissue condition and local expression levels of the signaling pathways. As a result of this, future studies on RAAS should focus less on a one peptide-one pathway approach, but on the evidence of tissue-specific pathways and on the local and systemic interactions of the RAAS. Furthermore, novel MasR agonists permit a new direction in RAAS-targeted therapy, and thus MasR shows a promising potential in future management of the global burden of HT, including the many associated comorbidities. Finally, this review emphasizes the need of studies to elucidate the signaling pathways of MasR, including the proposed cross-talks with additional RAAS components, and furthermore, the crucial need of clinical trials to confirm that the Mas-dependent pathway has a therapeutic effect in human essential HT. This requires demonstration of sufficient BP control and/or less adverse effects, in comparison with the use of classic RAAS inhibitors.
